# Multiplex serology for *Streptobacillus moniliformis* and other ‘rat bite fever-like’ microorganisms for seroprevalence studies in rodents

**DOI:** 10.1371/journal.pone.0333888

**Published:** 2025-10-15

**Authors:** Katja Schmidt, Julia Butt, Ulrich Matt, Klaus Vogel, Calvin Mehl, Rainer G. Ulrich, Tobias Eisenberg

**Affiliations:** 1 Microbiological Diagnostics, German Cancer Research Center, Heidelberg, Germany; 2 Infections and Cancer Epidemiology, German Cancer Research Center, Heidelberg, Germany; 3 Department of Medicine V, Internal Medicine, Infectious Diseases and Infection Control, Universities of Giessen and Marburg Lung Center (UGMLC), member of the German Center for Lung Research (DZL), Justus-Liebig University (JLU) Giessen, Giessen, Germany; 4 Institute of Novel and Emerging Infectious Diseases, Friedrich-Loeffler-Institut, Federal Research Institute for Animal Health, Greifswald - Insel Riems, Germany; 5 German Center for Infection Research (DZIF), Hamburg–Lübeck–Borstel–Riems, Greifswald–Insel Riems, Germany; 6 Department Veterinary Medicine, Hessian State Laboratory (LHL), Giessen, Germany; World Health Organization, Regional Office for South-East Asia, INDIA

## Abstract

Rat bite fever (RBF) is a zoonotic disease caused primarily by *Streptobacillus* (*S*.) *moniliformis*. Norway or brown rats (*Rattus* [*R.*] *norvegicus*) are the natural host for *S. moniliformis* and carry the bacterium in the nasopharynx without clinical disease. Transmission to humans often occurs through rat bites or scratches, but also through contact with the excreta of infected rats. Although human infections with *S. moniliformis* occur worldwide, they are rarely diagnosed. For decades, *S. moniliformis* was the only known member of the genus *Streptobacillus*. In recent years, however, four additional species were identified, two of which being zoonotic pathogens capable of causing symptoms identical to RBF in humans. The aim of this study was to develop a serological assay covering all known *Streptobacillus* species. A bead-based multiplex fluorescence immunoassay for *S. moniliformis* detection has been used for years in routine diagnostics of laboratory rodents. Here, this assay was adapted to enable the detection of antibodies against all currently known *Streptobacillus* species and tested with sera from experimentally inoculated mice and rats, and with negative sera from laboratory rodents. Using this assay, we broadly examined the prevalence of *Streptobacillus* spp. reactive antibodies in wild rodents. Transudates from a total of 107 Norway rats, 81 black or roof rats (*Rattus rattus*) and 110 house mice (*Mus musculus*) from different husbandries and wildlife populations within Germany were tested. Antibody prevalences of 41% in *R. norvegicus* and 83% in *R. rattus* suggest that *Streptobacillus* spp. are widespread in wild and captive rats in Germany, whereas wild mice seem to be free of infection. Due to its high throughput capacity and multiplex format, the *Streptobacillus* multiplex serology is well suited for large seroprevalence studies in rodents and has the potential, after adaptation, for use in humans, thereby allowing for the assessment of *Streptobacillus* infection risk and risk of RBF.

## Introduction

*Streptobacillus* (*S*.) *moniliformis* is the main causative agent of rat bite fever (RBF) and its food-borne variant, Haverhill fever [1–3]. RBF was reported for the first time in the United States in 1839 [4], but was not associated with a specific pathogen until 1914, when Schottmüller described *Streptothrix muris ratti*, isolated from a rat-bitten man [5]. In 1925, the organism was renamed *Streptobacillus moniliformis* [3]. RBF occurs worldwide and is believed to be under-reported and under-diagnosed in humans, most likely because of a lack of awareness of the disease among clinicians, indirect animal contact, the unavailability of reliable diagnostics, and because it is not a notifiable disease worldwide [2,6–8]. People with direct or indirect contact to rats are particularly at risk of infection: homeless people, farmers, hunters and trappers, veterinarians and their employees, pest controllers, sewer workers, pet shop personnel and pet owners [6]. Due to the increased popularity of pet rats, children are especially at risk of infection [2,6,7,9,10]. Untreated RBF can lead to serious health complications, including endo- and myocarditis, hepatitis, pericarditis, pneumonia, septicemia and meningitis, and even death [6,8,11–19].

Norway or brown rats (*Rattus norvegicus*) are the natural hosts of *S. moniliformis*, and typically carry the pathogen asymptomatically in their oro- or nasopharynx [2,20–22]. *Streptobacillus moniliformis* transmission to humans is most often reported after rat bites or scratches [6,11,12,14,23–27], but transmissions may also occur indirectly through contact with excreta or saliva of infected rats [2,28–30], or the consumption of contaminated food or drinking water [31,32]. *Streptobacillus moniliformis* has been found in wild rats [2,20,21], laboratory rats [33–35], pet (or fancy) rats [26,36,37], feeder rats [8], and has also been isolated from laboratory mice following natural transmission from rats or experimental infection [38–41]. Other rodent species (e.g., spinifex hopping mouse, gerbil, guinea pig, squirrels (*Paraxerus cepapi*, *Xerus erythropus*)), companion (dogs and cats) and exotic animal species (koalas and non-human primates), as well as livestock (turkey) were reported to be susceptible to infection and develop clinical disease [6,38,42–48]. However, in many of these studies, the isolates are no longer available or were later identified as novel taxa [49–51].

For a long time, the genus *Streptobacillus* was believed to be monotypic, comprising *S. moniliformis* as the only species of this genus. Since 2014, five novel species have been described: *Pseudostreptobacillus* (*P.*) *hongkongensis* [52,53], *Streptobacillus felis* [54], *Streptobacillus notomytis* [51], *Streptobacillus ratti* [55] and *Streptobacillus canis* [56]. *Pseudostreptobacillus hongkongensis* has been exclusively isolated from humans, and is considered to be part of the natural microbiota of the oropharynx [57]. *Streptobacillus notomytis,* originally isolated from a spinifex hopping mouse (*Notomys alexis*) [58], and *S. ratti* are closely associated with black or roof rats (*Rattus rattus*) [20,21,59]. *Streptobacillus felis* and *S. canis* are associated with cats (domestic cat, *Felis silvestris catus,* and rusty-spotted cat, *Prionailurus rubiginosus*) and dogs (*Canis lupus familiaris*), respectively [54,56,60]. To date, there are no comprehensive studies on the distribution or prevalence of these newly identified *Streptobacillus* species, and thus their significance as human pathogens remains unclear. However, recent studies have reported the isolation of *S. notomytis* and *S. felis* from human patients presenting with RBF-like symptoms [61–63].

The identification of the vast majority of human RBF cases is based on the detection of genetic material (by sequencing of 16S rRNA gene amplicons and other genes), whereas culture-based isolation of *S. moniliformis* from blood, joint-fluid, or rash-associated lesions is less frequent due to the organism’s extreme fastidiousness [1,23,64]. In contrast, the use of serological assays for the detection of *Streptobacillus*-specific antibodies has only been reported for a few human cases [65,66]. At present, *Streptobacillus*-specific serological assays are either not easily accessible to clinicians or simply unavailable. Consequently, serological testing is not included in the diagnostic workup of human disease cases [1]. In laboratory rodents, detection of *S. moniliformis* infections remains particularly relevant for health monitoring, as *S. moniliformis* was common in laboratory rats during the first half of the last century [67]. In mice, most documented outbreaks (epizootics) of streptobacillosis were linked to rats housed in the same room or in close vicinity, or due to *S. moniliformis* transmission via aerosols or handling by animal caretakers [68,69], although rats were not always identified as the source of infection [38–40,70]. Nowadays, *S. moniliformis* is extremely rare in laboratory facilities; nevertheless, *S. moniliformis* testing is still included in health monitoring programs of laboratory animal facilities housing rats and/or mice [71]. Serological tests are usually preferred for monitoring *S. moniliformis* infections in rodent colonies because serological detection methods are assumed to be more sensitive in detection than to culture methods [72]. Different serological tests, including indirect immunofluorescence assay (iIFA) [29,38], enzyme-linked immunosorbent assay (ELISA) [34,73] and immunoblot [33] have been described in the literature for serological testing in rats and mice. Serological assays for rodent-relevant *Streptobacillus* species other than *S. moniliformis* are neither commercially available nor reported in the literature to date.

A bead-based multiplex fluorometric antibody binding assay comprising *S. moniliformis* has been used for health monitoring of laboratory rodents at the Microbiological Diagnostics of the German Cancer Research Center (DKFZ) in Heidelberg, Germany, for years and is part of the routine multiplex serology for the detection of antibodies specific to murine-associated viruses and bacteria. Published examples include its application for detecting antibodies to murine astroviruses and *Helicobacter* species [74,75]. A multiplex serology approach is advantageous over other serological tests because of the potential to simultaneously detect antibodies against multiple infectious agents in a single reaction, the small sample volume needed, the high-throughput capability and resultant cost-effectiveness.

The objective of this study was to develop a sensitive and specific serological assay capable to detect antibodies to all known *Streptobacillus* species, with high-throughput capability for use in large-scale seroprevalence studies in rodents. The bead-based *S. moniliformis* serological assay used at the DKFZ in Heidelberg was adapted to the detection of antibodies to all other currently recognized *Streptobacillus* species and was tested using sera from experimentally inoculated mice and rats. The development of this high-throughput assay enabled the implementation of the first seroprevalence study in wild and captive (wild-caught) rodents demonstrating that *S. moniliformis* and *S. notomytis* are widely distributed in rats.

The *Streptobacillus* multiplex serological assay described in this study will enable large-scale seroprevalence studies in both animals and humans in the future, facilitating the assessment of the prevalence and potential drivers of infection with *Streptobacillus* species known to cause RBF-like disease.

## Materials and methods

### Origin and housing of laboratory mice and rats

CD1 (official strain nomenclature Crl:CD1 (ICR); Crl strain code 022) and BALB/c (BALB/cOlaHsd) mice were originally purchased from Charles River Laboratories (Sulzfeld, Germany) and Harlan Laboratories (Indianapolis, IN, USA; now Inotiv, West Lafayette, IN, USA), respectively. They were bred as gnotobiotic mice, colonized with the Taconic Altered Schaedler flora, for biotechnical and health monitoring purposes, and housed under high hygiene conditions in the central breeding unit at the vivarium of the DKFZ in Heidelberg. Mice between 5−8 weeks of age were transferred to the BSL-2 unit one week before inoculation and housed in individually ventilated cages (Greenline; Tecniplast, Buguggiate, Italy).

WISTAR rats between 4−5 weeks of age (official strain nomenclature RjHan:WI) were purchased from Janvier Labs (Le Genest-Saint-Isle, France) and kept for one week in the BSL-2 unit, in individually ventilated cages, for acclimatization before they were inoculated.

All cage beddings (aspen material), nesting material (aspen wood, 24–120 mm, Abedd Vertriebs GmbH, Vienna, Austria), food (Mouse and Rat Maintenance No. 3437, KLIBA NAFAG, Kaiseraugst, Switzerland) and water were autoclaved before use. Cage changing was done under a biosafety cage changing station (BS48; Tecniplast, Buguggiate, Italy). For infection and sampling, animals were handled in a class II biological safety cabinet (MaxiSafe202, Thermo Fisher Scientific Inc., Waltham, MA, USA; BSK, Weiss Technik GmBH, Reiskirchen, Germany). New overgloves were used after all mice or rats of the same infection group were handled, and 0.5% (v/v) Wofasteril (Kesla Hygiene AG, Bitterfeld-Wolfen, Germany) was used for disinfection after working processes.

In order to maintain the high hygienic status of mice in the central breeding unit, intensive routine hygiene monitoring was conducted by testing contact sentinel mice and colony animals weekly for viral and bacterial infections and parasites. Microbiological testing included all infectious agents listed by the Federation for European Laboratory Animal Science Associations (FELASA) [71] plus encephalomyocarditis virus (EMCV, species *Cardiovirus rueckerti*), murine astroviruses (MuAstV), mouse kidney parvovirus (MKPV, species *Chaphamaparvovirus rodent1*), yeasts and all aerobic growing bacteria cultured on Columbia sheep blood agar (PB5039A; Oxoid^TM^, Thermo Fisher Scientific Inc., Waltham, MA, USA) and chocolate agar (PO5090A; Oxoid^TM^) for 24 hours at 37°C followed by 48 hours at 37°C and 5% CO_2_. Microbiological testing of animals was conducted in the Microbiological Laboratory of the Center for Preclinical Research at the DKFZ using bacteriological methods, multiplex serology, iIFA, ELISA, hemagglutination inhibition assay, conventional (RT-)PCR, quantitative (q)PCR/RT-qPCR, multiplex PCR and microscopy. Mice and rats used in this study were free from all pathogens and opportunists listed by the FELASA, and EMCV, MuAstV and MKPV.

The health and behavior of laboratory animals were checked daily by visual inspection by experienced animal keepers. If any deviations from normal behaviour occured, i.e., possible signs of pain such as abnormal movements, a changed facial expression and suppressed normal behavior patterns, the experimenters, the responsible person on site (veterinarian) and the animal welfare officer were immediately notified, even at weekends or on public holidays. Moribund animals were euthanized immediately by the animal keepers. Animals with abnormal behavior were checked by the experimenters and, if necessary, animal welfare officer and euthanized immediately if humane endpoints were reached, but no later than one hour after notification.

The animal facility of the DKFZ has been officially approved by the responsible authority (Regional Council of Karlsruhe, Germany) under the official approval file no. Az. 35–9185.64BH DKFZ. Housing conditions are, thus, in accordance with the German Animal Welfare Act (TierSchG) and the EU Directive 2010/63/EU. Designated veterinarians according to Article 25 of Directive 2010/63/EU, and Animal-Welfare Body, according to Article 27 of Directive 2010/63/EU, ensured compliance with institutional guidelines and legal regulation regarding care and handling of animals. All persons who handle laboratory animals are obliged to complete a training course that imparts the knowledge and skills required for the care, euthanasia or use of animal in experiments, in accordance with Annex 1 of the Laboratory Animal Welfare Ordinance (TierSchVersV).

### Inoculation of laboratory mice and rats

#### Preparation of bacterial suspensions.

Subcultures of *Streptobacillus* type strains (*S. moniliformis* ATCC 14647^T^, *S. felis* 131000547^T^, *S. notomytis* AHL-370-1^T^, *S. ratti* OGS16^T^, *S. canis* IHIT1603-19^T^ and *P. hongkongensis* DSM26322^T^) were used to prepare McFarland standards of 1–2 McFarland units (MFU, approximately 3–6 x 10^8^ colony-forming units (CFU)/ml [76]) for oral and intranasal inoculation, and of 0.5–1 MFU (approximately 1.5–3 x 10^8^ CFU/ml) for intraperitoneal inoculation of laboratory mice and rats.

#### Inoculation of mice.

**Fig 1:**
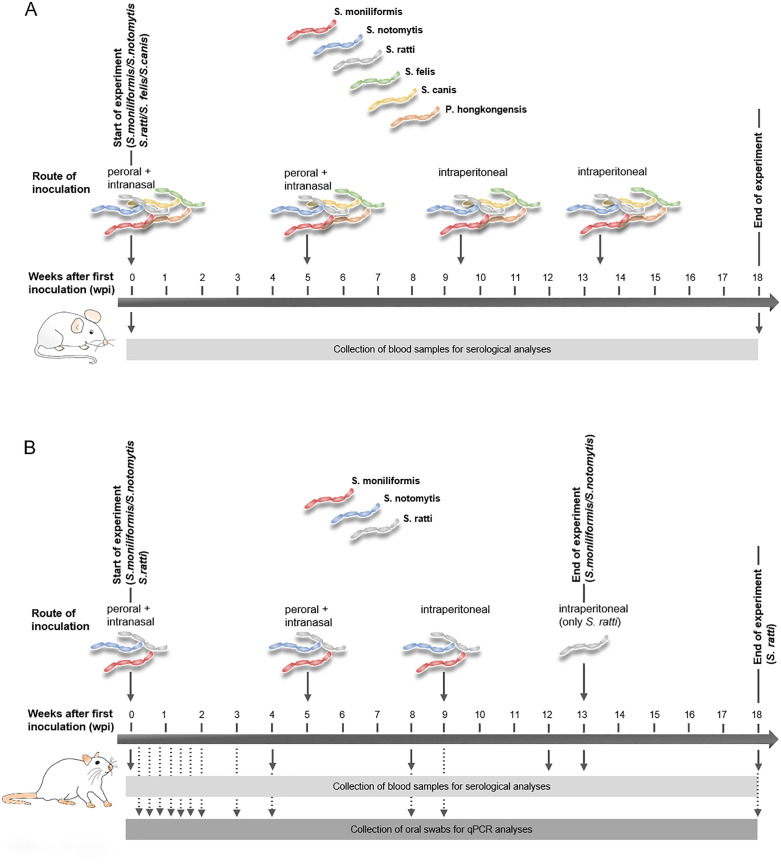
Schematic overview of the timeline of inoculation and sample collection in mice (A) and rats (B) inoculated with S*treptobacillus* spp. (A) Five CD1 mice per group were inoculated with *S. moniliformis*, *S. notomytis*, and *S. ratti*, while five BALB/c mice per group were used for inoculation with *S. felis*, *S. canis*, and *P. hongkongensis*. Each group received two inoculations via the oral and intranasal routes. This was followed by two intraperitoneal boosters. The experiment was terminated in week 18 after the first inoculation and blood samples were collected for serological analyses. (B) Four WISTAR rats per group were inoculated with *S. moniliformis*, *S. notomytis,* and *S. ratti*. Each group received two inoculations via the oral and intranasal routes. This was followed by one intraperitoneal booster with *S. moniliformis* and *S. notomytis*, and two intraperitoneal boosters with *S. ratti*. Oral swabs were collected for qPCR analysis on days 0, 2, 4, 6, 8, 10, 12, and 14, and in weeks 3, 4, 8, and 9 after the first inoculation. Blood samples were collected for serological analysis in weeks 0, 4, 8, and 12. The experiment was terminated in week 13 for the *S. moniliformis* and *S. notomytis* groups, and in week 18 for the *S. ratti* group. At termination, oral swabs, organs and blood samples were collected for both qPCR and serological analyses.

The inoculation protocol followed our established in-house inoculation procedure for generating positive sera. CD1 and BALB/c mice (five mice per group/*Streptobacillus* species) were inoculated orally and intranasally with 100 µl and 10 µl of bacterial suspensions (of either *S. moniliformis, S. felis, S. notomytis, S. ratti, S. canis* or *P. hongkongensis*), respectively (Fig 1A). Four to five weeks after the initial inoculation, mice were re-inoculated both orally and intranasally with a bacterial suspension as described above. About four weeks later, they received an intraperitoneal booster with 100 µl of a bacterial suspension, which was repeated once more after another four-week interval. Approximately four weeks following the final (fourth) booster, the animals were euthanized with CO_2_ using a GasDocUnit (medres medical research, Cologne, Germany), and blood was collected by cardiac puncture during dissection. Animals were monitored continuously throughout the experiment, and upon reaching one or more of the predefined endpoint criteria listed below, the experiment was terminated immediately and the animal was humanely euthanized without delay: piloerection, unphysiological, abnormal posture, apathy, hypothermia and cyanosis, auto-aggressiveness, automutilation, abnormal breathing difficulties and motor abnormalities (ataxia, reluctance to move, paralysis of the limbs). Blood samples were incubated at 4°C overnight and then centrifuged at 3,700 × *g* for 30 min before serum was separated from the blood clots, and kept at −20°C for further analysis. Sera were diluted 1:5 in hemagglutination (HA) buffer (90 mM NaCl, 3.45 mM NaH_2_PO_4_, 7 mM Na_2_HPO_4_, pH 7.2) before being further diluted in pre-incubation buffer (see below).

#### Inoculation of rats.

Before inoculation, blood samples and oral swabs were collected to be used as baseline samples for serological and qPCR analyses (Fig 1B). Four WISTAR rats per group/*Streptobacillus* species were inoculated orally and intranasally with 200 µl and 50 µl, respectively, of a bacterial suspension containing either *S. moniliformis, S. notomytis* or *S. ratti*. Four weeks after the first inoculation, blood samples were collected from the tail vein to evaluate the immune response by multiplex serology, and rats were again inoculated orally and intranasally as described above. Another four weeks later, blood was collected and rats were inoculated intraperitoneally with 200 µl bacterial suspension. Only for the *S. ratti* inoculated group, intraperitoneal inoculation was repeated four weeks later, because antibody levels were comparably low or even below the cut-off value. Oral swabs for qPCR analysis were collected every second day until day 14 post inoculation, and subsequently during weeks 3, 4, 8 and 9 post inoculation. Swab collection was performed exclusively in rats, as mice had previously been inoculated in accordance with the approved standard immunization protocol for the generation of positive sera. Due to insufficient antibody production against *S. ratti* in mice, inoculations were repeated in rats using a comparable protocol. This approach was extended to include the collection of oral swabs, which were subsequently analyzed by *Streptobacillus*-specific qPCR to evaluate the susceptibility of *R. norvegicus* to *R. rattus*-adapted *Streptobacillus* species. The difference between the mouse and rat inoculation is shown in Fig 1. Four weeks after the last inoculation, animals were euthanized with CO_2_ using a GasDocUnit (medres medical research, Cologne, Germany) and blood was collected by cardiac puncture during dissection. Oral swabs were taken and tissue samples from the tongue, lymph nodes, heart, liver, lungs, kidneys, spleen, small and large intestine were taken under sterile conditions. Fecal samples were collected from the rectum or anus. During dissection, internal organs and tissues were checked for gross pathological abnormalities. Tissue and organ samples were frozen at −20°C until further analysis by qPCR. Blood samples were incubated at 4°C overnight and then centrifuged at 3,700 × *g* for 30 min before serum was separated from the blood clots and kept at – 20°C for further analysis. Sera were diluted 1:5 in HA buffer (90 mM NaCl, 3.45 mM NaH_2_PO_4_, 7 mM Na_2_HPO_4_, pH 7.2) before being further diluted in pre-incubation buffer (see below).

#### Animal ethics.

Inoculation of mice for the generation of positive control sera in the frame of this study was officially approved by the local governmental authorities (Regional Council of Karlsruhe, Germany) under the notification number A-24/17 (approval date 2017-11-22). Inoculation of rats was performed under the notification number A-04/23 (approval date 2013-04-19).

### Collection of serum and transudate samples and sample preparation

#### Laboratory mice and rats.

To assess the specificity of the *Streptobacillus* multiplex serology, we used 44 serum samples of laboratory mice, two samples of multimammate mice (*Mastomys coucha*), one sample of CAST/EiJ (*Mus musculus castaneus*) and 34 serum samples of laboratory rats, which were microbiologically examined in the frame of routine health monitoring of rodents at the DKFZ. Of the laboratory mice, 23 samples originated from various transgenic mouse strains, 14 samples from Swiss Webster (Tac:SW) outbred mice, five from C57BL/6, and two from black nude mice (Supporting Information, S3 File). Almost all rat samples (except one) originated from Copenhagen (COP/CrCrl) rats, an inbred rat strain bred at the DKFZ. The negative *S. moniliformis* status of all laboratory rodents at DKFZ has continually been determined by routine microbiological testing at weekly intervals as recommended by the FELASA [71].

Blood samples were either collected from live animals by puncture of the tail vein or the Vena temporalis superficialis, or by cardiac puncture during dissection after euthanasia with CO_2_. After overnight incubation at 4°C and centrifugation, sera were separated and diluted 1:5 in HA buffer (90 mM NaCl, 3.45 mM NaH_2_PO_4_, 7 mM Na_2_HPO_4_, pH 7.2) before being further diluted in pre-incubation buffer (see below).

#### Wild and captive (wild) rodents.

Wild rodents were captured in the frame of various pest management programs conducted at livestock farms, zoological gardens, and other locations in Germany. Dead animals were immediately frozen and stored at −20°C until dissection. In addition to free-living wild rodents, samples were also collected from captive-bred wild rats. The term ‘captive wild rats’ refers to offspring from wild rats that were bred and kept in colonies in laboratory settings or large enclosures for several years or decades. All animals were dissected under BSL-3 conditions at the Friedrich-Loeffler-Institut, Greifswald-Insel Riems, Germany, and transudate (thoracic lavage) was obtained by rinsing the chest cavity with 1 ml phosphate-buffered saline (PBS; 136 mM NaCl, 2.6 mM KCl, 8.1 mM Na_2_HPO_4_ x 2H_2_O, 1.5 mM KH_2_PO_4_, pH 7.2–7.4). Transudates were directly diluted in pre-incubation buffer (see below) without previous dilution in HA buffer.

In total, 107 transudate samples were obtained from wild and captive Norway rats (*Rattus norvegicus*), 81 samples from wild and captive black rats (*Rattus rattus*), and 110 samples from wild house mice (*Mus musculus*)*.* If needed, species identification was based on cytochrome b mitochondrial DNA sequencing as described elsewhere [77]. Details on the habitat, husbandry and capture date as well as on their characteristics (sex and weight) are given in S3 File, Supporting Information.

#### Animal ethics.

Laboratory mice and rats were housed in individually ventilated cages (IVC; Greenline, Tecniplast, Buguggiate, Italy) at the vivarium of the DKFZ as given under ‘Origin and housing of laboratory rodents’. Captive wild rats were kept in animal facilities based on holding permits provided by the responsible veterinary or state office at the indicated date (Veterinary Office of the district of Potsdam-Mittelmark, 2014-06-23). Wild rodents were trapped by professional pest control operators in accordance with the regular pest-control treatments in Germany according to the German Infection Protection Act (IfSG §17). Hence, no capture permit was required.

### Preparation of bacterial membrane protein extracts

Bacteria (*Streptobacillus* species and non-target bacteria, i.e., *Aeromonas* (*A.*) *sobria*, *A. hydrophila* and *Bacillus* (*B.*) *thuringiensis* serovar Israelensis) were amplified in Standard-I nutrient broth (Merck KGaA, Darmstadt, Germany) or tryptic soy broth (Millipore, Merck KGaA) supplemented with 150 ml/l fetal calf serum or 200 ml/l horse serum, respectively, and microaerobic incubation for 1–2 days at 37°C until an optical density (OD) OD600 of approximately 1.0 was reached. The suspension was centrifuged at 4,000 × *g* at 4°C for 30 min. Supernatant was discarded and bacterial pellet was resuspended in a tenth of the original volume HA buffer, followed by three washings with HA buffer to remove traces of the cultivation media. The pellet was lysed with 1% (v/v) Octyl-β-D-glucopyranoside (O8001, Sigma-Aldrich, Merck KGaA) in HA buffer for one hour at room temperature. The lysate was again centrifuged at 4,000 × *g* at 4°C for 45 min, and the supernatant, containing bacterial membrane proteins, was carefully collected.

Slide-A-Lyzer dialysis cassettes 10K MWCO (no. 66810, Thermo Fisher Scientific Inc., Waltham, MA, USA) were equilibrated in HA buffer before the supernatant was injected into the dialysis cassette, and bacterial membrane proteins in the supernatant were dialyzed against HA buffer at a 250-fold excess in volume. Dialysis was performed at 4°C overnight (approx. 18 hours) and buffer was changed at least four times before the bacterial membrane lysates were collected and aliquots stored at −20°C for further use.

### Protein coupling to beads

The membrane protein extracts were loaded on individual sets of spectrally distinct polystyrene beads that contain embedded fluorescent dyes (MagPlex^®^ microspheres; Luminex, Austin, TX, USA). A published protocol [78] was slightly modified. Briefly, beads were resuspended and 100 µl bead suspension (approx. 1.25 × 10^6^ beads) was washed three times in the following sequence: centrifugation for 2 min at 20,000 × *g*, removal of the supernatant, addition of the activation buffer (0.1 mol/l sodium phosphate, pH 6.2), sonification for 1 min, and resuspension of the pellet by vortexing. Finally, beads were resuspended in 40 µl activation buffer and 5 µl 1-(3-Dimethylaminopropyl)-3-ethylcarbodiimide (EDC; 50 mg/ml in activation buffer; Thermo Fisher Scientific Inc., Waltham, MA, USA) and 5 µl N-hydroxysuccinimide (NHS; 50 mg/ml in dimethyl sulfoxide, DMSO; Thermo Fisher Scientific Inc.) were added before incubation for 20 min at room temperature in the dark on a horizontal shaker. The activated beads were washed three times with coating buffer (15 mmol/l Na_2_CO_3_, 35 mmol/l NaHCO_3_; pH 9.6) as described above.

Protein concentrations in bacterial membrane lysates were measured according to Bradford [79]. For protein loading on beads, lysates were diluted in coating buffer to a final concentration of 50 µg/ml. After the final washing step, beads were resuspended in 100 µl lysate (ratio of 1:1 of the original bead volume) and incubated overnight at 4°C in the dark on a horizontal shaker. After incubation and three washings with washing buffer (0.5 ml/l Tween 20 in 1 × PBS, pH 7.4), beads were resuspended in 100 µl saturated milk powder solution (100 g/l milk powder (AppliChem GmbH, Darmstadt, Germany) in washing buffer), vortexed and incubated for one hour at 4°C in the dark on a horizontal shaker. Thereafter, loaded beads were washed three times with washing buffer and resuspended in 50 µl storage buffer (1 g/l casein, 0.5 ml/l sodium azide in 1 × PBS; ratio of 0.5:1 of the original bead volume) and stored at 4°C in the dark.

### *Streptobacillus* multiplex serology

The multiplex serology is based on an immunosorbent assay in combination with the fluorescent bead technology from Luminex Corporation (https://int.diasorin.com/en/luminex-ltg). The *Streptobacillus* multiplex serology panel comprises *S. moniliformis*, *S. felis*, *S. ratti*, *S. notomytis*, *S. canis* and *P. hongkongensis*. Bacterial membrane proteins were extracted and directly coupled on polystyrene beads with distinct embedded fluorescent dyes (MagPlex^®^ Microspheres; Luminex Corp., Austin, Texas, USA) as described above.

General set-up and protocol of the multiplex serology is described elsewhere [78,80]. Sera and transudates were diluted 1:250 and 1:50, respectively, in pre-incubation buffer (1 g/l casein, 5 g/l polyvinylalcohol, 8 g/l polyvinylpyrrolidone, 2 g/l total protein lysate (from bacteria overexpressing Glutathione S-Transferase tag without adhering sequences to block binding of antibodies) in 1 × PBS) and incubated for one hour at room temperature to suppress unspecific binding of antibodies to the beads themselves [80]. The differently labelled and protein loaded beads were mixed to be incubated with an equal volume of diluted serum in a single reaction on a 96-well plate (final dilution 1:500 for mouse and rat sera and 1:100 for wild rodent transudates). The *Streptobacillus* multiplex assay was run as a six-plex assay for rat sera, and as a seven-plex assay for mice sera. In the latter, an immunoglobulin (Ig) G control, which allowed differentiation between immunocompetent and immunodeficient mice with reduced amounts of IgG in the serum, was included (bead set coated with anti-mouse IgG, AffiniPure donkey anti-mouse IgG, 4 µg/ml beads; cat. no. 715-005-150, Jackson ImmunoResearch Laboratories, Inc., West Grove, PA, USA). The Luminex analyzer BioPlex200 (BioRad Laboratories GmbH, Munich, Germany) was used to distinguish between the bead set, and consequently the bound *Streptobacillus* species membrane lysates, and to quantify the amount of bound serum antibody by a secondary antibody (biotinylated AffiniPure goat anti-mouse IgM + IgG (H + L; cat. no. 115-065-044, Jackson ImmunoResearch Laboratories), goat anti-rat IgM + IgG (H + L; cat. no. 112-065-068; Jackson ImmunoResearch Laboratories), 1:1000 diluted in blocking buffer (1 g/l casein in 1 × PBS)) and a fluorescent reporter conjugate (Streptavidin-R-Phycoerythrin, MOSS Inc., Pasadena, CA, USA; 1:500 diluted in blocking buffer). To assess background reactivity, all bead sets were additionally run in parallel without serum. Final antigen-MFI values were measured of at least 50 beads per bead set and sample; background reactivity was accounted for by subtracting the individual bead MFI values resulting from a serum-free reaction.

**Fig 2:**
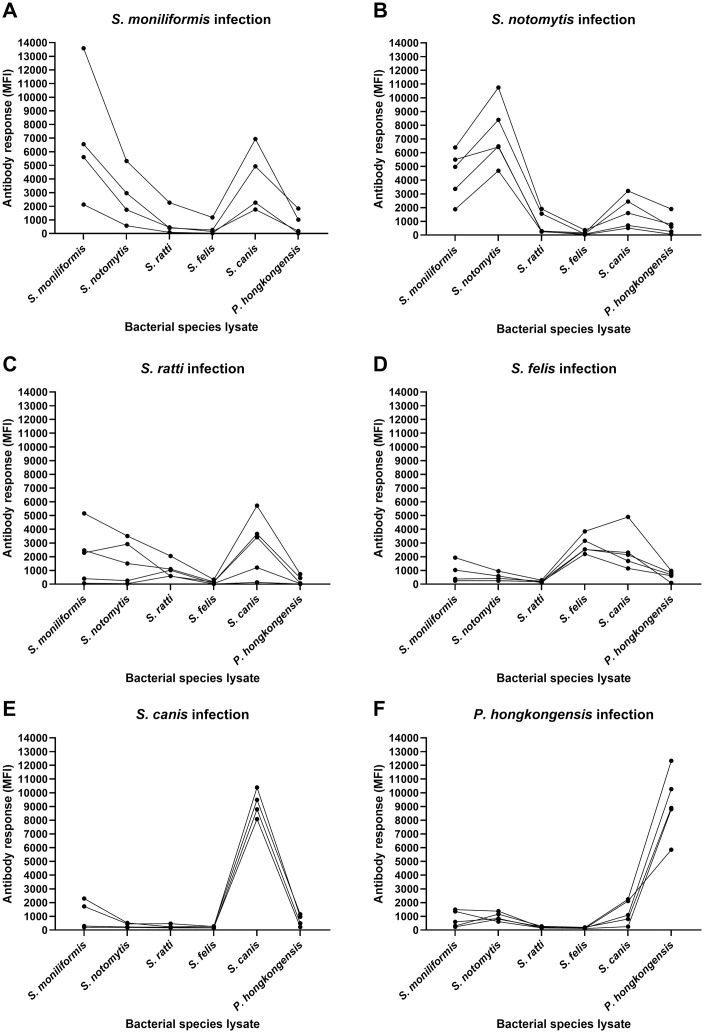
Antibody response in BALB/c and CD1 laboratory mice 18 weeks after inoculation with different *Streptobacillus* species. Antibody reactivities in terms of MFI values measured by *Streptobacillus* multiplex serology after inoculation with *S. moniliformis* in CD1 mice (A), *S. notomytis* in CD1 mice (B), *S. ratti* in CD1 mice (C), *S. felis* in BALB/c mice (D), *S. canis* in BALB/c mice (E) and *P. hongkongensis* in BALB/c mice (F) are shown. Each individual mouse was inoculated twice orally and intranasally, followed by two intraperitoneal inoculations. At the end of the experiment, all mice exhibited MFI values above the respective cut-off thresholds (i.e., *S. moniliformis*: 172, *S. notomytis*: 163, *S. ratti*: 80, *S. felis*: 136, *S. canis*: 114, *P. hongkongensis*: 109) and were classified as seropositive. Five mice per group were inoculated, however clinically diseased mice were taken out of the experiment and analysis: one *S. moniliformis* inoculated CD1 mouse due to hindquarter paralysis and one *S. canis* inoculated BALB/c mouse due to poor general condition. Connected data points correspond to an individual mouse.

**Fig 3:**
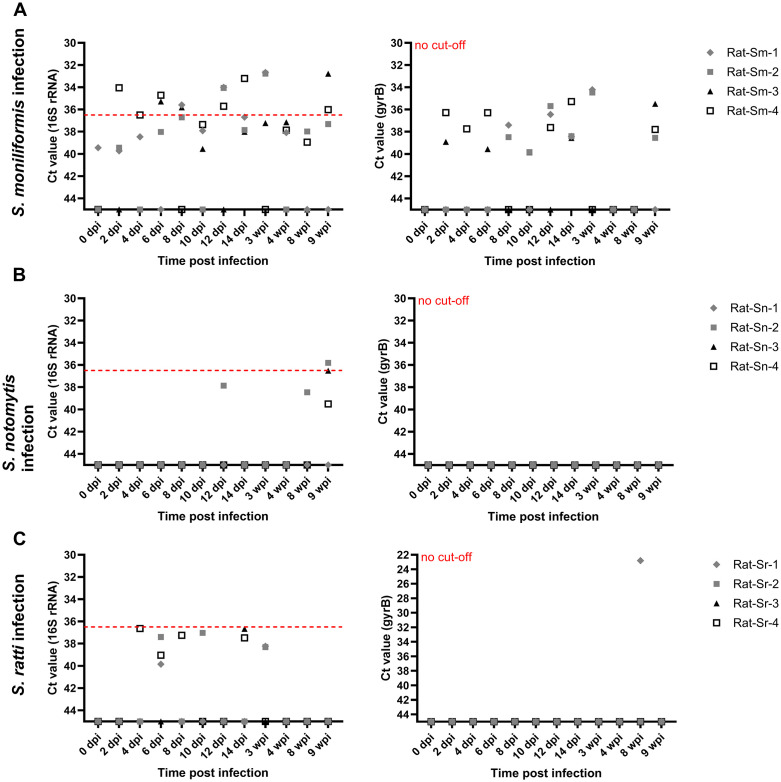
*Streptobacillus* specific qPCR targeting bacterial 16S rRNA gene (left graph) and *gyrB* gene (right graph) of oral swabs from *S. moniliformis* (Sm, A), *S. notomytis* (Sn, B) and *S. ratti* (Sr, C) inoculated rats. Cycle threshold (ct) values from oral swabs are plotted against timepoints of swab collection after inoculation (dpi, days after first inoculation; wpi, weeks after first inoculation) for four rats per group (Rat-Sm/Sn/Sr No. 1-4). The y-axis is given in reverse order to stress positive findings, i.e., ct values above the cut-off. The red horizontal dotted line indicates the qPCR assay-specific cut-off value (16S rRNA qPCR only).

**Fig 4:**
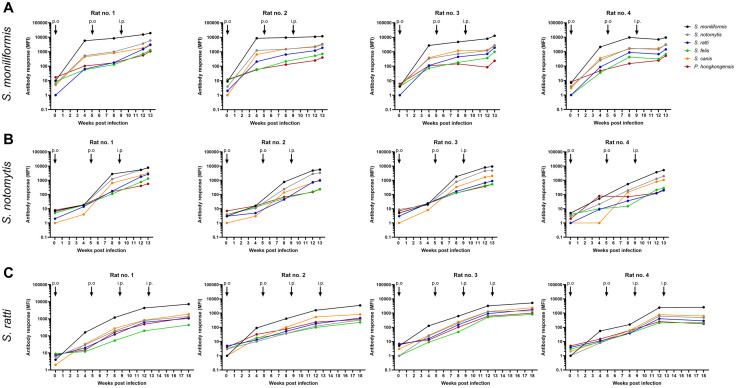
Antibody response after inoculation of WISTAR rats with *S. moniliformis* (A), *S. notomytis* (B) and *S. ratti* (C) over 13 or 18 weeks. Antibody reactivities in terms of MFI values measured by *Streptobacillus* multiplex serology after two oral and intranasal inoculations and an additional intraperitoneal injection (A and B) or two additional intraperitoneal inoculations (C) over 13 (A and B) or 18 weeks (C) are depicted. Connected data points correspond to the time course of reactions against one species-specific *Streptobacillus* membrane protein lysate. Arrows indicate the time points of peroral and intranasal (p.o.) and intraperitoneal (i.p.) inoculations.

**Fig 5:**
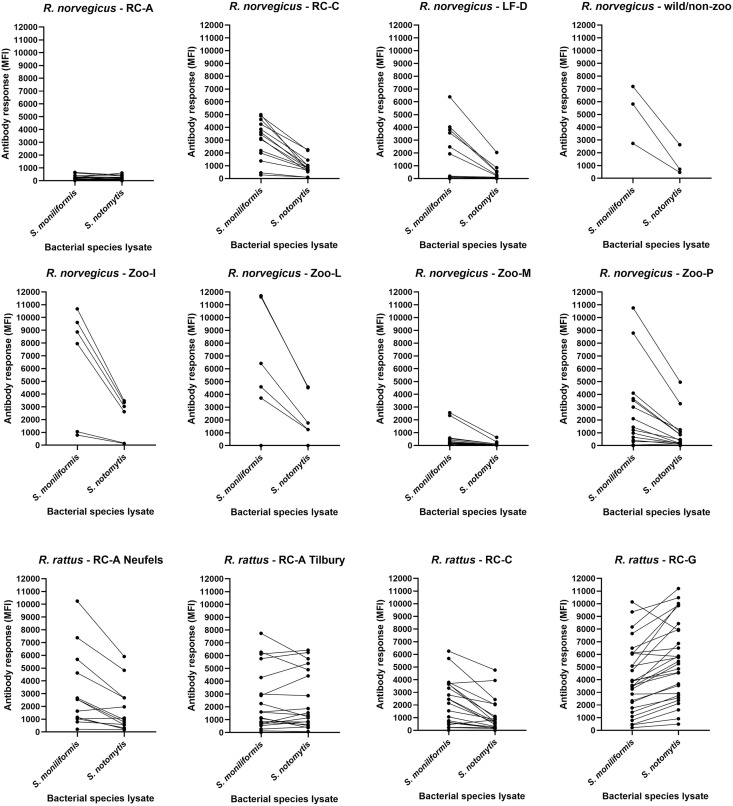
Reaction patterns of *S. moniliformis-* and *S. notomytis-*specific antibodies in Norway rats (*R. norvegicus*) and black rats (*R. rattus*) from different capture sites, or from wild rat-derived colonies. MFI values measured by *Streptobacillus* multiplex serology are shown. The dots represent data points; connected data points correspond to an individual rat.

GraphPad Prism version 10.2.0 (GraphPad Software, Boston, MA, USA) was used to create the figures (Figs 2–5).

### *Streptobacillus* qPCR analysis

A previously designed triplex qPCR assay, which showed high specificity for the detection and quantification of *S. moniliformis* in different clinical matrices, was employed for the detection of *Streptobacillus* spp. [22]. Briefly, automated DNA extraction from oral swabs and tissue samples obtained during necropsies was performed on IndiMag 48s (Indical Bioscience GmbH, Leipzig, Germany) using the Kylt^®^ RNA/DNA Purification HTP kit (SAN Group Biotech Germany GmbH, Höltinghausen, Germany) according to manufacturer’s instructions and samples stored at −20°C until further use. Primers Smoni-16S-F (5’-GGTTATCCCAGTCTAAGAGGTAAGTTCT-3’) and Smoni-16S-R (5’-AGAATGCTTAACACATGCAAATCTATG-3’), and probe Smoni-16S-P (5’-CY5-CACGTTACTCACCAGTCCACCATGTCTCTTATCT-3’) target the 16S rRNA gene of all known *Streptobacillus* species, while primers Smoni-gyrB-F (5’-AGTTTAAAATTCCCTGAACCACAATT-3’) and Smoni-gyrB-R (5’- ACTTCCAAACACTCCTGAAACTATACTTG-3’) and probe Smoni-gyrB-P (5’-FAM -TCACAAACTAAGGCAAAACTTGGTTCATCTGAG-3’) target the *gyrB* genes of *S. moniliformis*, *S. notomytis*, *S. ratti* and *P. hongkongensis* but not those of *S. canis* and *S. felis*. An internal control reaction was used as published by Hoffmann et al. using primers EGFP-11-F (5’-CAGCCACAACGTCTATATCATG-3’) and EGFP-10-R (5’-CTTGTACAGCTCGTCCATGC-3’) and the EGFP probe (5’-HEX AGCACCCAGTCCGCCCTGAGCA-3’ [81]). The qPCR was performed using the QuantiTect probe Mastermix kit (Qiagen, Hilden, Germany). Five µl extracted DNA were mixed with 12.5 µl mastermix, 0.2 µl of the enhanced green fluorescence protein (EGFP) coding plasmid DNA (2x10^5^ copies/µl) and water to give a total volume of 25 µl. AriaMx Real-time PCR System cycler (Agilent Technologies, Waldbronn, Germany) was used for amplification, and PCR conditions included an initial denaturation at 95°C for 900 s followed by 45 cycles of 94°C for 15 s and 60°C for 60 s. To distinguish between true and false positives for the 16S rRNA gene detection, a cut-off cycle threshold (ct) value of 36.5 was adopted, so that only samples with a ct value below 36.5 were considered positive.

## Results

### Development of a serological multiplex assay for *Streptobacillus* spp. specific antibody detection

Membrane protein lysates of all currently known species of the genus *Streptobacillus*, i.e., *S. moniliformis*, *S. notomytis*, *S. ratti*, *S. felis*, *S. canis* and *P. hongkongensis*, were prepared to be used as antigens in a fluorescent bead-based multiplex serology assay. Membrane protein lysates of non-target species were included in the multiplex serology to examine possible cross-reactions to membrane proteins of other bacterial species, i.e., *A*. *sobria*, *A. hydrophila* and *B*. *thuringiensis* serovar Israelensis.

To evaluate the specificity of the assay, sera from 45 laboratory mice and two multimammate mice (n = 47) and rats (n = 34) from the DKFZ animal facility were used as a negative sample set. In this negative sample set, the background reactivities to *S. moniliformis*, being the most relevant *Streptobacillus* species for laboratory rodents, as an example, were low with a median fluorescence intensity (MFI) of 24 (interquartile range, IQR: 8.75–54.25) in mice and 157 (IQR: 121.63–303.38) in rats (Supporting Information, S1-C and S2-B Figs). Because a larger sample set of mice and rats with a defined positive anti-*Streptobacillus* antibody status and a valid reference (gold standard) assay were not available, the cut-off values were calculated by the mean value of reactivities of the negative sample set plus three-times the standard deviation (mean + 3x SD) [78,82–84], as given in Table 1.

**Table 1:** Calculated cut-off median fluorescence intensity (MFI) values for the different *Streptobacillus* species based on mean values of the negative sample set + three standard deviations.

	cut-off MFI	
	mice	rats
*S. moniliformis*	172	686
*S. notomytis*	163	458
*S. ratti*	80	138
*S. felis*	136	159
*S. canis*	114	214
*P. hongkongensis*	109	1,024

### Inoculation of laboratory mice with different *Streptobacillus* species

To assess the sensitivity of our assay, BALB/c mice were inoculated (experimentally infected) twice by oral and intranasal route followed by two intraperitoneal injections with the different *Streptobacillus* species. BALB/c mice were chosen for the generation of positive sera due to their resistance to clinical disease following *S. moniliformis* infection and their capacity to mount a strong antibody-mediated immune response [38,73]. Because inoculation with *S. notomytis* and *S. ratti* was not successful in BALB/c mice, i.e., there was no antibody production induced, the inoculation was repeated in CD1 mice. Inoculation with *S. moniliformis* was performed only in CD1 mice. In one of five *S. moniliformis* inoculated CD1 mice, infection caused bilateral hindquarter paralysis resulting in the termination of the experiment and euthanasia of the mouse for ethical reasons. *Streptobacillus*-specific quantitative (q)PCR of DNA isolated from joint tissue was negative and histopathological analysis of the mouse did not reveal any infection-related alterations. *Streptobacillus notomytis* infection caused weight loss and swelling of the tarsal joint in three, and unilateral hindquarter paralysis in one, of five BALB/c mice, resulting in the termination of the experiment, whereas the infection in CD1 mice was subclinical. *Streptobacillus*-specific qPCR of the joints yielded negative results. One of five *S. canis* infected mice showed a poor general condition and was thus taken out of the experiment.

As shown in Fig 2, inoculation resulted in varying antibody levels measured by *Streptobacillus* multiplex serology with median MFI values of 6,109 (IQR: 4,767–8,339) in *S. moniliformis* inoculated CD1 mice, 6,512 (IQR: 6,463–8,440) in *S. notomytis* inoculated CD1 mice, 1,056 (IQR: 628 − 1,133) in *S. ratti* inoculated CD1 mice, 2,599 (IQR: 2,585 − 3,217) in *S. felis* inoculated BALB/c mice, 9,161 (IQR: 8,636–9,734) in *S. canis* inoculated BALB/c mice, and of 9,129 (IQR: 9,036–10,499) in *P. hongkongensis* inoculated BALB/c mice, referring to the respective *Streptobacillus* species bead set. MFI values of individual mice were always above the calculated cut-off value for the respective *Streptobacillus* species, resulting in 100% seropositive mice (Supporting Information, S2 File). However, cross-reactivities in the *Streptobacillus* multiplex serology varied. While homologous reactions to *S. canis* lysate and *P. hongkongensis* lysate were specific, sera from *S. moniliformis* inoculated mice showed cross-reactivity with *S. notomytis* and *S. canis* lysates. Vice versa, *S. notomytis* inoculated mice displayed a high cross-reactivity with *S. moniliformis* lysate, presumably due to their close genetic relationship (Supporting Information S3 Fig). Sera from *S. ratti* inoculated mice yielded significant reactivities with lysates of *S. moniliformis* and *S. canis*, but not with *S. ratti* lysate. Finally, *S. felis* inoculated mice showed comparable reactivities with lysates of both closely related *Streptobacillus* species, *S. felis* and *S. canis*.

### Inoculation of rats and infection course of rat-specific *Streptobacillus* species

*Streptobacillus* species which cause natural infection of rats, namely *S. moniliformis, S. notomytis* and *S. ratti*, were also used for inoculation of laboratory rats (*R. norvegicus*). In contrast, *S. felis*, *S. canis*, and *P. hongkongensis* were not re-inoculated into rats, as rats are not their natural hosts and no additional scientific insight was anticipated from their inclusion. Analysis of oral swabs, taken over the first nine weeks of the experiment, by qPCR targeting sequences within the 16S rRNA gene and the *gyrB* gene, revealed that a reliable detection of *Streptobacillus* spp. DNA was not possible. Only oral swabs from *S. moniliformis* inoculated rats tested positive by both qPCRs, although individual animals did not consistently test positive, and qPCR signals were often below the cut-off value, as shown in Fig 3. Oral swabs from *S. notomytis* and *S. ratti* inoculated rats only rarely yielded positive signals and most ct values in the 16S rRNA gene qPCR were below the cut-off value.

All samples recovered during dissection, i.e., oral swab, tongue, lymph nodes, heart, liver, lungs, kidneys, spleen, small and large intestine and feces, tested negative by *Streptobacillus* specific qPCR, except the lymph node of one *S. moniliformis* infected rat, which yielded a positive result with a ct value of 36 in both qPCR assays, i.e., for 16S rRNA and *gyrB* genes (see Supporting Information, S1 File).

Blood samples were collected from all rats before, and 4, 8 and 12 weeks after the first inoculation to evaluate the success of inoculation in terms of antibody production. Fig 4 depicts the course of antibody production over time after inoculation with different *Streptobacillus* species. Experimental *S. moniliformis* infection yielded high antibody levels within four weeks after the first oral and intranasal inoculation. In contrast, in *S. notomytis* and *S. ratti* inoculated rats, a second round of inoculation resulted in low antibody levels, only slightly above the cut-off value in individual rats. Because comparably high antibody titer levels were found in *S. moniliformis* and *S. notomytis* inoculated rats after the first intraperitoneal inoculation (booster), a second intraperitoneal injection was omitted and animals were dissected 13 weeks after the first inoculation. Final antibody titers were relatively high with a median value of 12,356 MFI (IQR: 11,322–14,461) in *S. moniliformis* inoculated rats and of 4,122 MFI (IQR: 3,021–5,537) in *S. notomytis* inoculated rats. Only the *S. ratti* inoculated rats received a second intraperitoneal injection of *S. ratti* because *S. ratti*-specific antibodies, measured 8 weeks after the first inoculation, were comparably low. Nonetheless, the final median MFI value 18 weeks after the first inoculation was comparably low at 752 MFI (IQR: 410–1,229). Nevertheless, MFI values of all rat sera, including *S. ratti* inoculated animals, were above the calculated cut-off values, thus all rats were considered seropositive, resulting in a sensitivity of 100% (Supporting Information, S2 File). Most remarkably, cross-reactivities with membrane protein lysates from non-target *Streptobacillus* species were high in all rats, with reactivities to *S. moniliformis* membrane lysates consistently being the highest. The *S. moniliformis* reactions (median MFI values) were 3.5 times higher than the reactions to *S. notomytis* in *S. moniliformis* inoculated rats. However*,* in *S. notomytis* inoculated rats, cross-reactive *S. moniliformis* antibody reactions were also 1.58 times higher than specific reactions to *S. notomytis*. Inoculation of rats with *S. ratti* yielded weak *S. ratti* specific reactivities in the multiplex serology but strong reactions with *S. moniliformis* membrane lysates, with a median MFI of 4,361 (IQR: 3,255 − 5,738).

### *Streptobacillus* seroprevalence in wild rodents

The *Streptobacillus* multiplex serology based on combined bacterial membrane protein lysate coupled bead sets was subsequently used to detect antibodies in wild rodents. Transudates (thoracic lavages) from wild rodents were collected as part of various pest control programs in livestock farms and zoos, and from captive wild rat colonies. A total of 107 samples from Norway rats, 81 samples from black rats and 110 samples from house mice from nine trapping sites or husbandries within Germany were analyzed by multiplex serology for evidence of a current or previous *Streptobacillus* infection. Table 2 summarizes data of wild mice and rats (wild and captive wild) included in this study, including their habitat, location (capture site) and references (if animals were used in previous publications), as well as numbers of antibody positives, median MFI values and interquartile range. Only the results of the *S. moniliformis* and *S. notomytis* serological assays are given in Table 2 because these are the relevant species in wild rats and mice in central Europe. Antibody responses to the different *Streptobacillus* species in wild and captive wild rats in comparison to laboratory rats are shown in S1 Fig, Supporting Information. Although cross-reactivities were observed, antibody responses were always highest with rat-associated *S. moniliformis* and *S. notomytis*, while MFI values with the other *Streptobacillus* species were consistently lower.

**Table 2:** Overview of wild rodents included in this study, antibody prevalence, antibody levels and testing characteristics.

							*S. moniliformis*		*S. notomytis*	
Species (total number)	Wild/ captive	Habitat	Location Code	Strain	Reference	No. animals tested	Median MFI (IQR)	% Sero-positive (no.)	Median MFI (IQR)	% Sero-positive (no.)
***Rattus norvegicus*** (n = 107)	captive	rat colony	RC-A	BSR	[85]	10	19 (13, 24)	0.0 (0)	15 (7, 17)	0.0 (0)
			RC-A	Lyon	[85]	6	166 (151, 173)	0.0 (0)	79 (64, 144)	0.0 (0)
			RC-A	MHS	[85]	10	98 (46, 118)	0.0 (0)	113 (41, 246)	10.0 (1)
			RC-A	WPHR	[85]	9	181 (54, 272)	0.0 (0)	74 (62, 489)	0.0 (0)
			RC-C		[86]	14	3290 (2045, 4148)	85.7 (12)	823 (615, 1021)	85.7 (12)
	wild	livestock farm	LF-D		[86]	14	167 (90, 3296)	42.9 (6)	85 (18, 448)	28.6 (4)
		pest rodents zoo	Zoo-I		[87]	6	8410 (2782, 9423)	100.0 (6)	2830 (781, 3275)	66.7 (4)
			Zoo-L			6	5506 (3930, 10319)	83.3 (5)	1509 (1250, 3835)	83.3 (5)
			Zoo-M		[87]	14	239 (119, 509)	14.3 (2)	41 (20, 104)	7.1 (1)
			Zoo-P		[87]	15	1441 (537, 3601)	66.7 (10)	390 (148, 964)	46.7 (7)
		Wild, non-zoo	different locations		partly referenced [87]	3	5815 (4272, 6504)	100.0 (3)	710 (587, 1668)	100.0 (3)
***Rattus rattus*** (n = 81)	captive	rat colony	RC-A	Neufels	[85,86]	13	2543 (1046, 4623)	92.3 (12)	954 (553, 2660)	76.9 (10)
			RC-A	Tilbury	[85,86]	20	1366 (674, 3324)	70.0 (14)	1291 (612, 4540)	80.0 (16)
			RC-C		[86]	20	2301 (620, 3641)	70.0 (14)	657 (250, 1338)	65.0 (13)
		zoo feeder rats (rat colony)	RC-G		[59]	28	3714 (2108, 6039)	89.3 (25)	5356 (2862, 7916)	100.0 (28)
***Mus musculus*** (n = 110)	wild	pest rodents zoo	Zoo-I		[87]	1	1 (1, 1)	0.0 (0)	1 (1, 1)	0.0 (0)
			Zoo-P			49	2 (1, 9)	0.0 (0)	4 (1, 15)	0.0 (0)
			Zoo-Q		[88]	60	1 (1, 6)	0.0 (0)	2 (1, 7)	0.0 (0)

RC – rat colony; LF – livestock farm; IQR – interquartile range; MFI – median fluorescence intensity; no – number.

None of the 110 wild mice, captured during pest control in three zoos in Germany, showed seropositive reactions. Overall MFI values were even lower than the MFI values measured in *Streptobacillus* infection-free laboratory mice (negative sample set) (Supporting Information, S2 Fig).

Transudate samples from 107 Norway rats from two captive rat colonies, four zoological gardens, a livestock farm and other capture sites showed a median MFI of 315 (IQR: 90–3,290), while transudates from 81 black rats from three captive rat colonies showed a median MFI of 2,031 (IQR: 635 − 4,900), demonstrating that *Streptobacillus* infections are widespread in wild and captive wild rats in Germany. Reactions were considered *Streptobacillus*-specific because high antibody levels did not correlate with high reactivities to other, non-target bacterial membrane lysates, i.e., lysates from *A. sobria*, *A. hydrophila* and *B. thuringiensis* serovar Israelensis. These bacteria are not members of the rat microbiota and only rarely yielded positive findings in individual rats (Supporting Information, S3 File).

The overall prevalence of *S. moniliformis* antibodies in Norway rats was 41% (n = 44) with MFI values as high as 11,700 being found in seropositive rats. Seroprevalence rates were highly dependent on the origin of the rat. With regard to *S. moniliformis*, the species associated with Norway rats, antibodies were detected in rats of most capture sites with seroprevalence rates ranging from 14% of rats from Zoo-M (n = 14) to 100% of rats from Zoo-I (n = 6). In contrast, all rats from four different wild-rat derived strains from location RC-A (n = 35) were negative (Table 2). However, it must be noted that the number of samples from the different populations varied considerably, ranging from one to 60 transudate samples.

In black rats, the overall *Streptobacillus* seropositivity was higher than in Norway rats. Of 81 serologically tested black rats, 83% (n = 67) had *S. notomytis* reactive antibodies. Due to high cross-reactivity between *S. moniliformis* and *S. notomytis*, seroprevalence calculations, based on *S. notomytis* reactive antibodies in Norway rats and *S. moniliformis* reactive antibodies in black rats instead, resulted in prevalence rates of 35% (n = 37) in Norway rats and 80% (n = 65) in black rats.

In seropositive wild rats from different populations, differing levels of cross-reactivity were observed with other *Streptobacillus* species. Most surprisingly, the observed pattern, i.e., the ratio of *S. moniliformis* to *S. notomytis* antibody levels, was highly variable depending on the origin of the rat (Fig 5). In Norway rats *S. moniliformis* reactivity (MFI values) was always higher than that of *S. notomytis*. In black rats, however, the natural reservoir of *S. notomytis*, antibody reaction patterns (i.e., ratios of *S. moniliformis* to *S. notomytis* MFI values) between the three capture or housing sites showed remarkable differences. While rats of strain Neufels and captive rats from RC-C showed lower reactivities with *S. notomytis* lysate than with *S. moniliformis* lysate (Neufels strain, median MFI 954 versus 2,543; RC-C, median MFI 657 versus 2,301), the opposite trend was observed in zoo feeder rats from RC-G (median MFI 5,356 versus 3,714). Rats of strain Tilbury showed similarly high cross-reactivities to *S. notomytis* and *S. moniliformis* (median MFI 1,291 versus 1,366) (Table 2).

## Discussion

This study describes the development of a serological assay for the detection of *Streptobacillus* spp. specific antibodies in mice and rats, and its application in the first large-scale seroprevalence study of wild and captive rodents. To date, RBF is not a notifiable disease worldwide, and no approved diagnostics are currently available; consequently, reliable prevalence data do not exist. In addition, no seroprevalence studies on *Streptobacillus* have been conducted in humans and those in animals have been limited to small-scale investigations. The paucity of such studies explains why a fundamental understanding of the infection course, the risk of infection, and the incidence of RBF is largely absent. The results of our study are intended to contribute to a better understanding of a group of overlooked and underdiagnosed zoonotic pathogens causing RBF or RBF-like disease.

### Development of a novel *Streptobacillus* multiplex serological assay

Following the recent description of four out of five new *Streptobacillus* species, new diagnostic tools are needed to detect and differentiate *Streptobacillus* infections in animals and humans. The objective of this work was the development of a serological assay with high-throughput capacity for *Streptobacillus* diagnostics that allows detecting antibodies to different *Streptobacillus* species in parallel. A microsphere-based multiplex fluorescent antibody-binding assay was chosen since it combines several benefits. Due to its ability to multiplex and monitor antibodies against multiple pathogens simultaneously in a single sample, the high sensitivity, high sample throughput and small sample volumes required, this assay is highly advantageous in health monitoring of laboratory rodents and large seroprevalence studies [89]. The *Streptobacillus* multiplex serological assay developed in this study utilizes membrane protein extracts coupled to polystyrene beads with distinct embedded fluorescent dyes, allowing simultaneous detection of antibodies against distinct *Streptobacillus* species in a single assay. This novel diagnostic tool allows the expansion of diagnostic panels to include additional, previously undetected rodent-associated *Streptobacillus* species, both for health monitoring of laboratory rodents and for seroprevalence studies in wild rodents.

### Varying antibody responses to different *Streptobacillus* species in mice and rats following inoculation

Our study presents for the first time experimental inoculations of mice and rats with different *Streptobacillus* species. To date, experimental infections with *Streptobacillus* species other than *S. moniliformis* have not been described, whereas numerous studies have documented both natural and experimental *S. moniliformis* infections in laboratory rodents [34,35,38,40,90–96]. Here, BALB/c mice were chosen for the generation of positive sera because they are usually resistant to clinical disease caused by *S. moniliformis* infection [38,73]. Because inoculation of BALB/c mice with *S. notomytis* and *S. ratti* did not elicit adequate antibody titer production, the inoculation was repeated in outbred CD1 mice, with limited success. Although all mice were seropositive at the end of the experiments, *S. ratti* specific antibody levels were comparably low. Based on experimental infection studies reported in the literature, it can be concluded that laboratory mice and rats develop an immune response within one to four weeks following oral, intranasal, intramuscular or intravenous challenge with *S. moniliformis* [33,34,70,72,73,92,97]. In mice, there are significant mouse strain-specific differences in seroconversion rates, composition, titer of antibodies and clinical signs, but dose, age and strain specific characteristics of *S. moniliformis* were also noted [33,38,70,72,73,92].

Moreover, in this study, experimental inoculation with the rat-adapted *Streptobacillus* species was also conducted in laboratory rats to assess seroconversion and antibody production, and to investigate the early stages of infection. Since *S. moniliformis* is especially adapted to Norway rats, experimental infection of the laboratory rats yielded high levels of specific antibodies within four weeks of the first oral and intranasal inoculation. In rats inoculated with *S. notomytis* or *S. ratti*, after a second round of inoculation, low antibody levels (slightly above the cut-off value) were found in a few of the inoculated animals. In rats inoculated with *S. notomytis*, only after intraperitoneal injection (booster) high antibody titers were detected. Although all rats were seropositive at the end of inoculation experiment, titers of *S. ratti-*specific antibodies in *S. ratti* inoculated rats were low, though slightly above the cut-off value. This may be because laboratory rats are Norway rats, the natural host for *S. moniliformis* but not for *S. notomytis* and *S. ratti* [21,59], but may also be explained by technical reasons, i.e., low accessibility of relevant epitopes (antigenicity) of the *S. ratti* protein membrane lysate*.*

### Limited diagnostic sensitivity of molecular detection of *Streptobacillus* infection in inoculated rats

Oral swabs obtained from experimentally inoculated rats in this study did not consistently test positive for *Streptobacillus* DNA by qPCR. This may be due to technical reasons as oral swabs taken from live animals are not necessarily of consistent quality and often do not provide sufficient material to overcome the threshold and yield a positive qPCR result. On the other hand, fluctuations in pathogen load and shedding may also be a reason for inconsistent detection. However, since high antibody levels were detected in all rats using *Streptobacillus* multiplex serology, this suggests that serological testing may be superior to molecular methods for routine health monitoring of rodents, offering greater diagnostic sensitivity. This is in line with experiments made by Boot and co-authors who found that more animals, experimentally infected mice and rats as well as wild rats, tested positive by ELISA than by culture methods [72]. PCR detection in rats was described to be most successful in tissue samples from pharynx and mandibular lymph nodes, salivary glands, trachea and spleen three to seven days after infection [98]. In this study, at the end of the inoculation experiment, almost all organs, tissues and swabs from the inoculated rats tested negative by qPCR. Based on our data, it cannot be excluded that the infection was cleared at the end of the experiment, resulting in a negative qPCR result. However, existing literature suggests that *Streptobacillus* bacteria are commensals and permanently colonize the oropharynx of their host. It is, therefore, more likely that qPCR detection failed due to low gene copy numbers below the detection limit, indicating that (q)PCR testing is not a reliable method for the proof of an infection.

### High antibody prevalence in wild and captive wild rats, but not in mice

The newly developed high-throughput *Streptobacillus* multiplex serological assay was applied to detect *Streptobacillus-*specific antibodies in wild rodents, captured in the frame of pest control programs, and in captive wild-caught rodent colonies. Although serological assays for *S. moniliformis* detection in laboratory rodents have been used for many years, screening in wild rodents has not yet been done on a broad scale [72]. In accordance with the literature, which suggests that house mice are not the natural hosts for *S. moniliformis* nor other *Streptobacillus* species [6], none of the 110 wild mice in this study showed seropositive reactions. In Norway rats, however, *Streptobacillus* antibody prevalence rates varied greatly between populations of different origins ranging between 0% and 100%. Since the number of samples from the different capture and husbandry sites varied, the seroprevalence rates given in this study are only approximations of the true prevalence in the respective populations. The absence of antibodies in wild rats from RC-A was attributed to the isolated husbandry of captive rat colonies, which prevented contact with other wild rodents and thereby impeded the spread of infections to naïve animals within the colony. In contrast, captured wild Norway rats from RC-C yielded a prevalence rate of 86%, indicating a high level of *S. moniliformis* circulation within this population. The seroprevalence of *S. notomytis* in captive wild black rats was generally higher, with seropositivity rates between 65% and 100%. This is in line with findings of a study by Julius and co-authors from South Africa who found higher *Streptobacillus* prevalence rates in black rats than in Norway rats [21]. Although *S. moniliformis* has yet to be isolated from black rats, and *S. notomytis* yet to be detected in Norway rats, varying antibody reaction patterns observable in rats of different origin raise the question if this phenomenon is attributable to a co-circulation of *S. moniliformis* and *S. notomytis* or to genetic differences of the rat populations and associated differences in antibody responses. The *S. moniliformis* serological assay of this study was previously used in a study on zoonotic pathogens in rats from urban areas in the Netherlands [99]. While positive PCR findings were rare, an overall seroprevalence rate of 33% was found in wild Norway rats (n = 407) collected in Amsterdam and Rotterdam [99], which corresponds with a seropositivity rate of 31% in Norway rats in Germany, described in this study. Due to the limited availability of high-throughput serological diagnostic tools, field prevalence studies have predominantly relied on PCR-based methods rather than serological testing [20,100]. Using PCR, previous studies have reported *S. moniliformis* prevalence rates as high as 50–100% in wild rats in the USA, South Africa and Japan [2,20,21,101]. PCR-based molecular testing, however, may result in an underestimation of *Streptobacillus* prevalence [22].

### Technical aspects and limitations of the *Streptobacillus* multiplex serology

Due to the lack of knowledge about immunogenic *Streptobacillus* proteins, membrane protein extracts were used in the *Streptobacillus* multiplex serology in order to detect antibodies specific to the different *Streptobacillus* species. The use of bacterial membrane protein lysates, however, poses notable challenges related to variability in antigen composition and concentration across different batches. These inconsistencies are primarily due to differences in bacterial culture conditions, variations in bacterial growth rates that can affect the expression levels of surface antigens, and the efficiency of lysis procedures. Such batch-to-batch variations can affect the antigenicity of the lysates, which ultimately affects the reproducibility and reliability of the serological test. Therefore, the application of standardized protocols and rigorous quality control measures is essential. The use of protein membrane lysates in serological assays, in order to confirm the microbiological status of laboratory rodent colonies, had been described earlier for *S. moniliformis* and other bacteria, e.g., *Helicobacter hepaticus* [72,73,102]. The use of *S. moniliformis* membrane proteins instead of whole bacterial cells was shown to reduce the background reactions in an ELISA, and to increase its specificity [1]. However, the multiplex serology described in this study did not allow for unequivocal determination of the underlying *Streptobacillus* species, or to differentiate infections caused by different *Streptobacillus* species, due to high cross-reactivity. The reason for this is most likely the use of membrane protein lysates, and the very close genetic/antigenic relationship between *Streptobacillus* species (S3 Fig), resulting in the formation of antibodies recognizing similar or even identical protein structures. It needs to be mentioned that the antigenicity of the individual bacterial lysates relative to each other was unknown. Varying levels of reactions to non-target *Streptobacillus* species may be, at least in part, a phenomenon induced through different grades of accessibility of antigen determinants (epitopes), and not only through specific versus non-specific reactions. The greatest cross-reactivity was observed in laboratory mice and rats, and in wild rats, inoculated with *S. moniliformis* or *S. notomytis*, which correlates with the close genetic relationship between *S. moniliformis*, *S. notomytis* and *S. ratti* [103]. Similarly, no antigenic differences could be observed between *S. notomytis* and *S. moniliformis* in immunoblot assays [33]*,* although *S. notomytis* displayed a unique profile in electrophoretic protein patterns [104]. Most remarkably, the extent of cross-reactions differed between host species and was different in experimentally infected (inoculated) mice and rats. Since it became obvious that serological testing is not conclusive, additional tests such as culture, matrix assisted laser desorption/ionization – time of flight (MALDI-TOF) mass spectrometry analysis or DNA sequencing may be required to confirm and identify the infectious agent. However, at least in rats, the underlying *Streptobacillus* species may be identified from the host-pathogen species association. For seroprevalence studies in wild rats, the *Streptobacillus* multiplex serology can easily be down-scaled to be used as a triplex serology (covering all rat-relevant *Streptobacillus* species, i.e., *S. moniliformis, S. notomytis, S. ratti*) or a duplex serology (without *S. ratti,* which yet occurs in Japan only). The serological examination of companion animals (dogs and cats), other wild animals, or humans was not part of this study. Therefore, the assessment of the immunogenic quality of *S. felis, S. canis* and *P. hongkongensis* membrane protein lysates is still in question, and the *Streptobacillus* multiplex serology needs to be tested for sera from these host species. In mice, however, infection with these *Streptobacillus* species resulted in specific antibody responses.

## Conclusions

In summary, our data demonstrate that *S. moniliformis* and *S. notomytis* are widespread in wild and captive wild rat populations with seroprevalence rates up to 100%. Because of the close association of rats with humans, particularly in urban environments, and the possibility of transmission even by indirect contact to rats [105–107], there is a considerable risk for acquiring *Streptobacillus* infection and RBF. It is, therefore, of fundamental importance that clinicians are aware of this zoonotic pathogen and the disease-related symptoms. Concerning the zoonotic potential of *Streptobacillus* species other than *S. moniliformis*, there are several reported cases of RBF in humans from Japan and Germany, caused by *S. notomytis* and *S. felis* [61–63]. The serological assay presented here can be adapted to detect *Streptobacillus*-specific antibodies in animals other than rodents, e.g., in cats and dogs, and in humans. It has already been used successfully for the diagnosis of a clinical case of a confirmed RBF patient with fever in Germany, arthralgia and endocarditis, and allowed for the differentiation of IgM and IgG antibodies [108]. Furthermore, it was also used in other serological studies [62,99]. Due to its multiplex format and high-throughput capability, it provides a useful and cost-effective tool for large-scale seroprevalence studies of pets, wild animals and humans in the near future. In conclusion, with the provision of a new diagnostic tool to assess *Streptobacillus* infections, this study provides insight into the distribution of *Streptobacillus* infections in rodents from Germany, and, in this context, highlights the risk of *Streptobacillus* infection and RBF for humans.

## Supporting information

S1 Fig Comparison of *Streptobacillus* specific antibody responses in wild and captive wild Norway rats (*R. norvegicus*) and wild captive black rats (*R. rattus*), and in laboratory rats (*R. norvegicus*) by multiplex serology. Dotplots of net MFI values are shown. The dots represent data points. Lines inside the boxes indicate the median values and whiskers the interquartile range (IQR, Q1-Q3), representing 50% of data. The y-axis is logarithmically scaled.
(TIF)

S2 Fig Comparison of *Streptobacillus* specific antibody responses in wild house mice capture in zoological gardens (A) and laboratory mice (B) analyzed by multiplex serology. Dotplots of net MFI values are shown. The dots represent data points. Line inside the boxes indicate the median values and whiskers the interquartile range (IQR, Q1-Q3), representing 50% of data. The y-axis is logarithmically scaled.
(TIF)

S3 Fig Phylogenetic tree based on the sequence of 742 core genes of *Streptobacillus* species. Sequences of type strains were downloaded from NCBI (accessions are given in brackets) and uniformly annotated with Prokka v1.14.6. To identify core genes, a pangenome analysis using Roary v3.13.0 was utilized applying soft thresholds of 50% identity and 50% coverage to account for species differences. *Pseudostreptobacillus hongkongensis* was used as an outgroup. The tree was calculated with Fasttree v2.1.11 applying a generalized time-reversible model and visualizations were drawn with iTol (https://itol.embl.de/).
(TIF)

S1 File Spreadsheet with qPCR results of organ- and tissue material and oral swabs from laboratory rats inoculated with *S. moniliformis*, *S. notomytis* and *S. ratti.* These data were used to generate Fig 3.
(XLSX)

S2 File Spreadsheet with multiplex serology results (MFI values) from laboratory mice and rats inoculated with different *Streptobacillus* species. These data were used to generate Figs 2 and 4.
(XLSX)

S3 File Spreadsheet with multiplex serology results (MFI values) from wild rodents (Norway rats, black rats and house mice) and from laboratory mice and rats. Details on the habitat of wild rodents, husbandry and capture date as well as on their characteristics (sex and weight) are given. These data were used to generate Fig 5, Table 2, and S1 and S2 Figs.
(XLSX)
