# Supplementary figures and images for: Multiplex serology for Streptobacillus moniliformis and other ‘rat bite fever-like’ microorganisms for seroprevalence studies in rodents
Source: PLoS One. 2025 Oct 15;20(10):e0333888. doi: 10.1371/journal.pone.0333888 (PMC12527138; doi:10.1371/journal.pone.0333888)

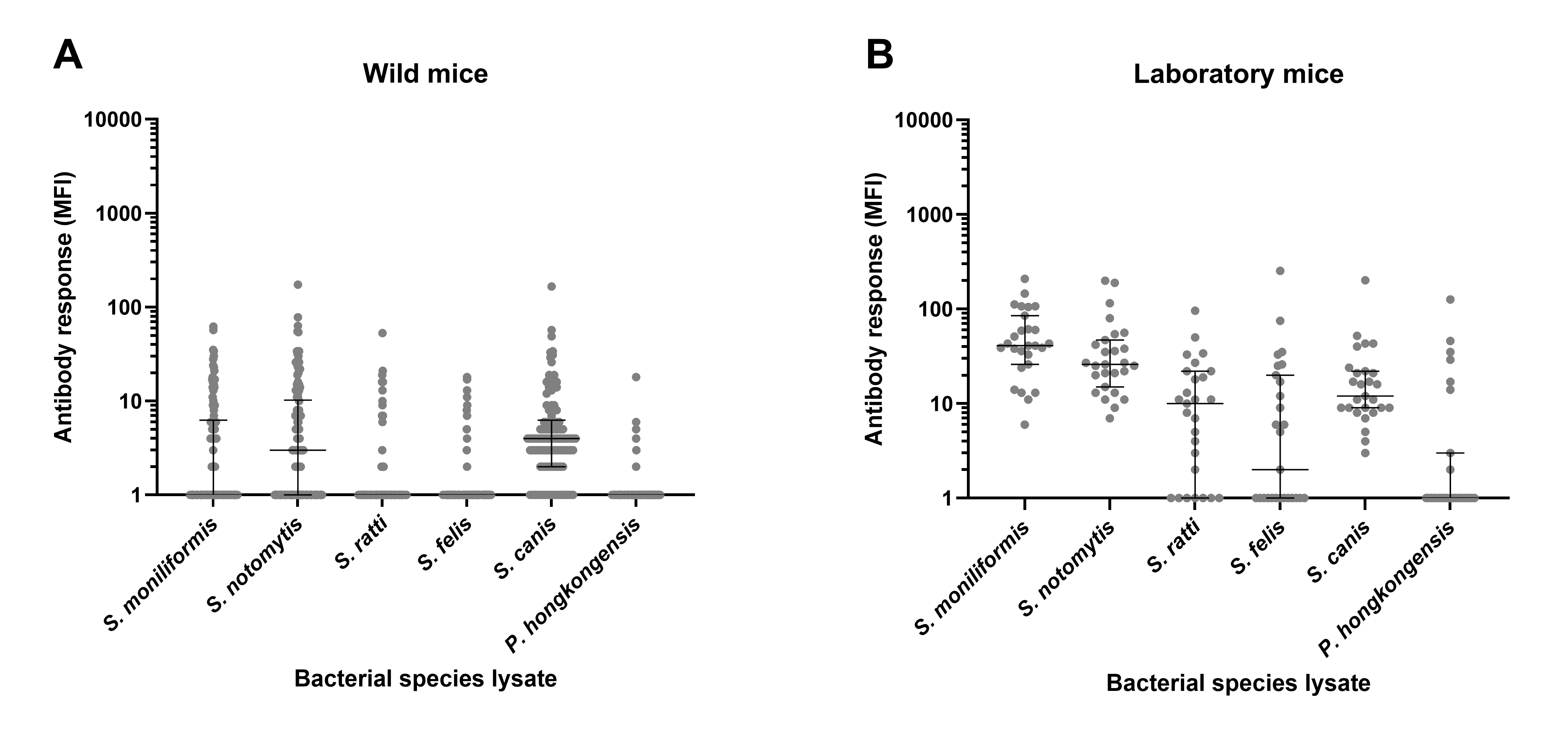

Supplement: S1 Fig — Dotplots of net MFI values are shown. The dots represent data points. Lines inside the boxes indicate the median values and whiskers the interquartile range (IQR, Q1-Q3), representing 50% of data. The y-axis is logarithmically scaled. (TIF) [file pone.0333888.s001.tif]

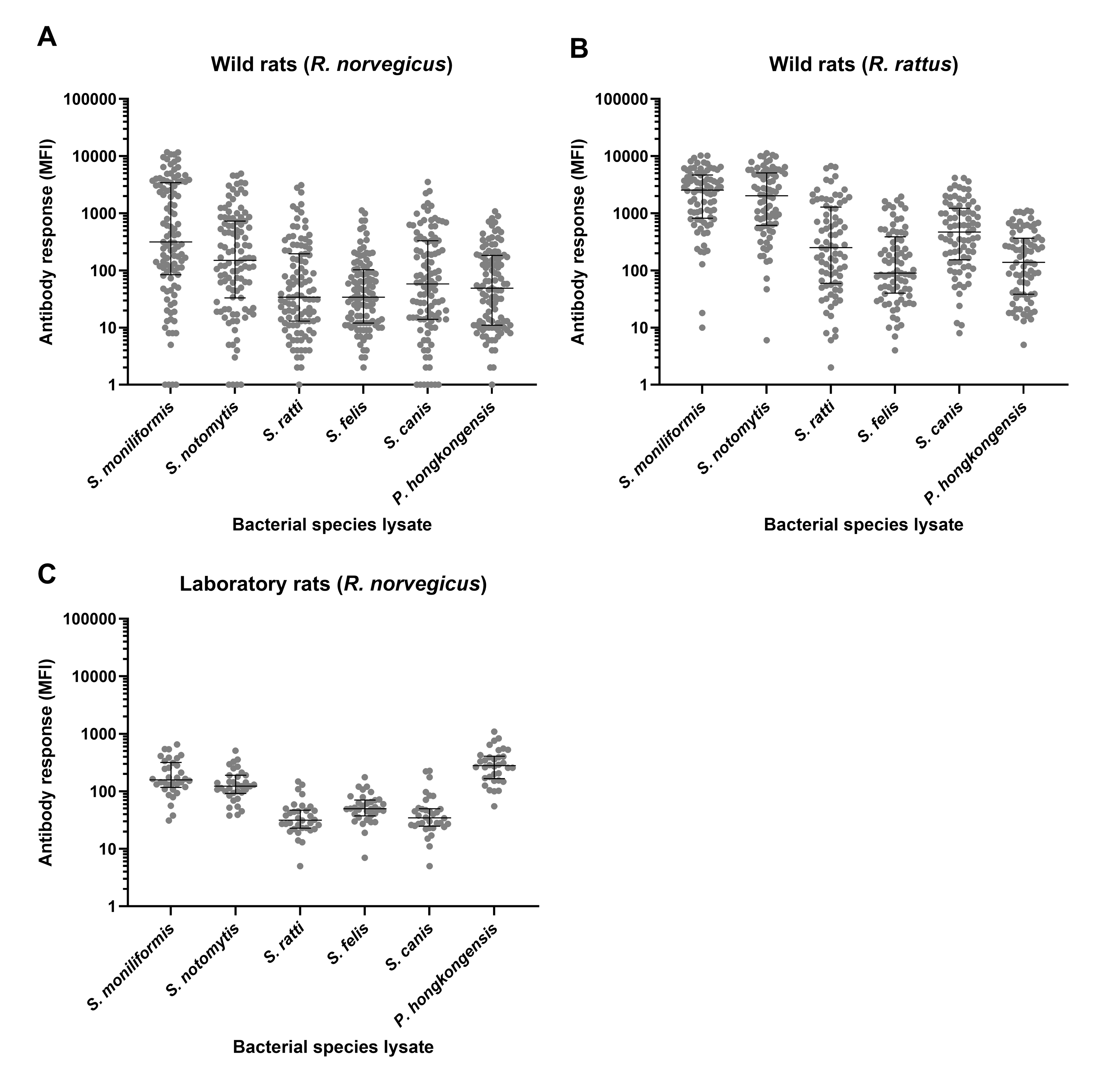

Supplement: S2 Fig — Dotplots of net MFI values are shown. The dots represent data points. Line inside the boxes indicate the median values and whiskers the interquartile range (IQR, Q1-Q3), representing 50% of data. The y-axis is logarithmically scaled. (TIF) [file pone.0333888.s002.tif]

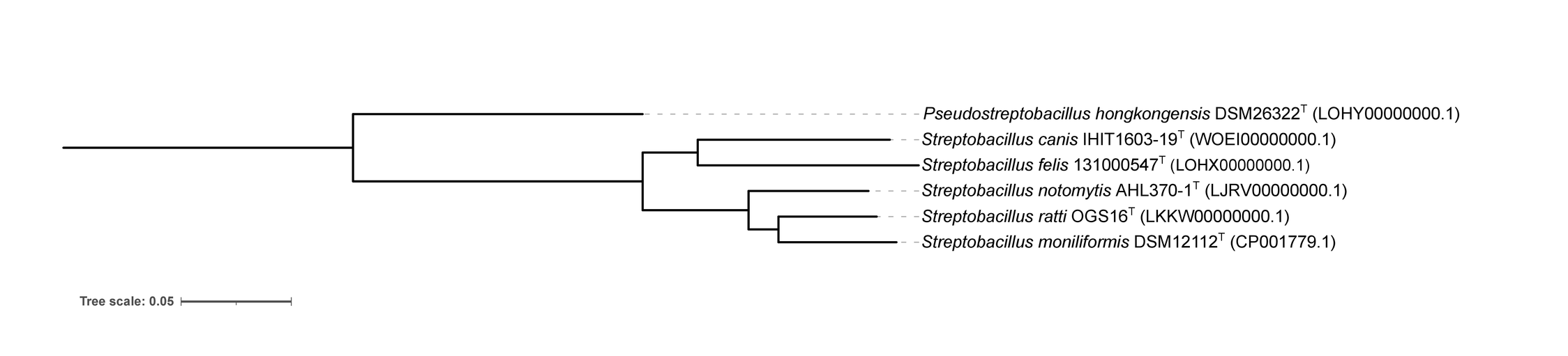

Supplement: S3 Fig — Sequences of type strains were downloaded from NCBI (accessions are given in brackets) and uniformly annotated with Prokka v1.14.6. To identify core genes, a pangenome analysis using Roary v3.13.0 was utilized applying soft thresholds of 50% identity and 50% coverage to account for species differences. Pseudostreptobacillus hongkongensis was used as an outgroup. The tree was calculated with Fasttree v2.1.11 applying a generalized time-reversible model and visualizations were drawn with iTol (https://itol.embl.de/). (TIF) [file pone.0333888.s003.tif]
